# Editorial: Advances in the therapeutic targeting of human matrix metalloproteinases in health and disease

**DOI:** 10.3389/fmolb.2023.1150474

**Published:** 2023-03-10

**Authors:** Shiv Bharadwaj, Amaresh Kumar Sahoo, Umesh Yadava

**Affiliations:** ^1^ Laboratory of Ligand Engineering, Institute of Biotechnology of the Czech Academy of Sciences, v.v.i., BIOCEV Research Center, Vestec, Czech Republic; ^2^ Department of Applied Sciences, Indian Institute of Information Technology Allahabad, Prayagraj, Uttar Pradesh, India; ^3^ Department of Physics, Deen Dayal Upadhyaya Gorakhpur University, Gorakhpur, India

**Keywords:** matrix metalloproteinases, extracellular matrix, therapeutics, diseases, molecular modeling & simulation

Matrix metalloproteinases (MMPs) are multi-domain proteins, including three conservation regions: pro-MMP, catalysis, and hemopexin, and belong to the large metzincin superfamily such as astacins, reprolysins, serralysins, and adamalysins or disintegrin metalloproteinases (ADAMs) (Loffek et al., 2011). Typically, MMPs are secreted by different tissues and cells, such as fibroblasts, endothelial cells, macrophages, neutrophils, *etc.*, detailed reviewed elsewhere (Cui et al., 2017). Herein, MMPs as proenzymes are produced or anchored to the cell surface and assembled in the cytoplasm in conjunction with a cytoplasmic domain and need extracellular activation followed by catalytic activities to proteins within the secretory pathway or extracellular space (Xie et al., 2017; Pehrsson et al., 2021). Thus, MMPs act as essential regulators for several active biomolecules, including chemokines, growth factors, proinflammatory cytokines, angiogenesis, cell proliferation and migration, wound healing, apoptosis and other physiological and pathological processes, summarized elsewhere (Malemud, 2006; Khalil, 2017; Mortensen et al., 2019; Laronha and Caldeira, 2020).

In the classical view, the activities of most MMPs are very low or negligible and are modulated by the activation of inhibitory precursor zymogens and tissue inhibitors of metalloproteinases (TIMPs) (Page-McCaw et al., 2007; Swarnakar et al., 2011; Khalil, 2017). Additionally, MMPs expression are transcriptionally controlled by inflammatory cytokines, growth factors, hormones, and cell–cell and cell–matrix interaction, detailed and reviewed elsewhere (Loffek et al., 2011). Under normal physiological conditions, MMPs can contribute by several mechanisms such as degradation of growth factors to make their availability to the distant cells that lack direct contact, digestion of various extracellular matrix (ECM) components and non-matrix proteins for the free transition of founder cells across the tissues into the adjacent stroma, and modulated restriction of receptors or biomolecules to terminate the invasive cell signaling pathways and cell migration, detailed reviewed elsewhere (Chang and Werb, 2001; Khalil, 2017). However, the dysregulation of MMPs’ biological function has been associated with the progression and development of various diseases, which can be categorized into three groups: (1) tissue demolition, (2) fibrosis, and (3) matrix deterioration, and role of MMPs in several diseases, including tumor metastasis and invasion, is also recently summarized by Helena et al. (Laronha and Caldeira, 2020). MMPs dysregulation causing tissue demolition may result in the severe diseases, such as ulcers, periodontal diseases, and cancer metastasis. For example, in hepatocellular carcinoma (HCC), MMP-10 was noticed to promote expression of the SDF-1 receptor and CXCR4, by involving reactive oxygen species (ROS) generation and mediation of hypoxia-inducible factor (HIF)-1α mediated CXCR4 promoter transactivation, which suggested to stimulate HCC cell proliferation, angiogenesis, and metastasis (Garcia-Irigoyen et al., 2015). Also, Emily et al. analyzed the 24 MMPs dysregulation in fifteen different cancer types to assess their diagnostic and prognostic potential revealing both consistent enhanced and decreased expression of MMPs across cancers (Gobin et al., 2019). Likewise, altered MMPs activities have been noticed to cause liver cirrhosis, fibrotic lung disease, otosclerosis, atherosclerosis, and multiple sclerosis while dilated cardiomyopathy, aortic aneurysm, and varicose veins are also demonstrated as results of the distorted functional role of MMPs. For example, in the case of idiopathic pulmonary fibrosis (IPF), MMP-3 and MMP-7 were noticed for promoting the epithelial-to-mesenchymal transition (EMT), MMP-3, MMP-7, and MMP-8 causes to enhance the activity of profibrotic mediators or decreasing the levels of antifibrotic mediators in lungs; and stimulation of fibrocyte migration by MMP-8 (Craig et al., 2015). Additionally, dysregulated MMPs, especially MMP-3 and MMP-12, were reported for the proteolytic cleavage of anti–tumor necrosis factor (anti-TNF) agents, *viz.* Infliximab, Adalimumab, and Etanercept, that resulted in the loss of function to neutralize tumor necrosis factor (TNF) as a treatment for Crohn’s disease (Biancheri et al., 2015). However, activities of MMP-2, MMP-9, and MMP-12 induced degradation of intestinal collagen levels have been suggested as a biomarker to predict the response of anti–TNF therapy in patients diagnosed with Crohn’s disease (van Haaften et al., 2020; Alexdottir et al., 2022). Interestingly, the administration of Adalimumab, the first fully human recombinant immunoglobulin G1 monoclonal antibody against soluble and membrane-bound tumor necrosis factor, was noted to substantially reduce the activity of inflammatory MMP-1 and MMP-9 while stimulating the production of wound-healing MMP-13 and tissue inhibitor of MMP-2 levels in the patients with hidradenitis suppurativa disease (Cao et al., 2021). Thus, MMPs have been in the scope of investigation under pharmaceutical drug discovery as drug-target for more than half a century, and the search for useful inhibitors is already ongoing for decades for linked diseases or disorders. For example, Nam et al. reported the synthetic antibody carrying extended CDR-H3 segments that efficiently and selectively inhibited the MMP-14 (Nam et al., 2016), and others designed and studied under clinical trials are reviewed elsewhere (Santamaria and de Groot, 2019; Laronha et al., 2020; Li et al., 2020). However, the designing and the development of MMPs inhibitors remain a great challenge because of low specificity and selectivity, instability, and observed side effects in clinical trials, reviewed elsewhere (Laronha et al., 2020; Li et al., 2020).

Therefore, this Research Topic aims to collect articles that highlight the promising strategies for the identification of molecular factors and their respective inhibitors, such as small molecular weight drug molecules, engineered proteins, clinical antibodies, *etc.*, driving the human extracellular Matrix or MMPs associated diseases and disorders in humans. This Research Topic, entitled “Advances in the Therapeutic Targeting of Human Matrix Metalloproteinases in Health and Disease” is composed of three original articles and two reviews and aims to demonstrate the essential role of MMPs in different diseases in humans and the identification of therapeutics agents to halt the MMPs-associated pathogenesis. In the original article, Xiao et al. (Fang et al., 2022) aimed to study the critical role of MMP-8, known for enhancing expression and linked with poor prognosis of sepsis in patients. Based on bioinformatics and experimental data analysis, the results marked the *MMP-8* as a vital gene in sepsis development while sepsis serum encouraging leukocyte adhesion to Human umbilical vein endothelial cells (HUVECs) *via* MMP-8, supports MMP-8 as a potential therapeutic target for the treatment of sepsis (Fang et al., 2022). Moreover, overexpression of MMP-9 essentially contributes to the development of cancer by supporting cancer cell invasion, tumor metastasis, and prompting the “angiogenic switch” (Gialeli et al., 2011; Jiang and Li, 2021). Thus, in absence of any clinically approved MMP-9, Shalini et al. (Mathpal et al.) used a deep learning algorithm and other complex computational techniques to find the novel MMP-9 inhibitors, which can simultaneously target Zn^2+^ and the catalytic pocket of MMP-9 enzyme (Mathpal et al.). The collected results established the substantial stability and affinity of only four hits in the active site of MMP-9. Notably, these compounds were reported for their therapeutic application in diseases while one compound was found as an experimental MMP-9 inhibitor. Collectively, they suggested the potential of computational pipeline in the identification of novel MMP-9 inhibitors while identified hits were advocated for the designing of lead compounds to treat cancer or to act as suitable drug candidates against MMP-9 (Mathpal et al.). Jun et al. (Yang et al.). Developed a model using the required information from the TCGA database which can correlate the glycolysis-related gene signature with the typical characteristics of tumor immune microenvironment in hepatocellular carcinoma (HCC) for the prediction of overall survival (OS) in cancer patients. In the reviews, Sabeena et al. (Mustafa et al.) summarized the multifunctional role of MMPs in the development of various cancers and discussed the various types of reported therapeutic inhibitors that can function against MMP’s activity. Finally, Georgina et al. (Gonzalez-Avila et al.) explored and discussed the novel MMPs-linked nanotechnology applications in which MMPs can be used for theragnostic purposes and as potential therapeutic targets to modulate the progression of cancer in patients.

Conclusively, MMPs have been studied for their altered functional activities contributing to the onset of multifactorial diseases and disorders in humans and thus, being has been in the spotlight of the pharmaceutical industry as a potential drug target. As numerous studies have established the essential role of MMPs in various biological systems, such as signal transmission and substrate recognition, and protein-protein interactions, non-specific or enhanced inhibition of a particular MMP may exert negative effects on the disease progression or the normal function of a biological system. For instance, musculoskeletal syndrome (MSS) was the most frequent side effect exhibited by synthetic inhibitors of MMPs in clinical trials (Georgiadis and Yiotakis, 2008; Jacobsen et al., 2010). Therefore, further understanding the role of multi-domain MMPs during the initiation, progression, and remission of associated diseases or disorders will assist both researchers and clinicians in the designing and synthesis of novel MMPs therapeutic blockers or inhibitors.
